# MeCP2 Deficiency Leads to Loss of Glial Kir4.1

**DOI:** 10.1523/ENEURO.0194-17.2018

**Published:** 2018-02-19

**Authors:** Uri Kahanovitch, Vishnu A. Cuddapah, Natasha L. Pacheco, Leanne M. Holt, Daniel K. Mulkey, Alan K. Percy, Michelle L. Olsen

**Affiliations:** 1 Department of Cell, Developmental and Integrative Biology, University of Alabama at Birmingham, Birmingham, AL 35294; 2 Department of Pediatrics, Civitan International Research Center, University of Alabama at Birmingham, Birmingham, AL 35294; 3 Virginia Tech School of Neuroscience, Blacksburg, VA 24061; 4 Department of Physiology and Neurobiology, University of Connecticut, Storrs, CT 06269, USA

**Keywords:** Epigenetic regulation, Kcnj10, MeCP2, Rett syndrome

## Abstract

Rett syndrome (RTT) is an X-linked neurodevelopmental disorder usually caused by mutations in methyl-CpG-binding protein 2 (MeCP2). RTT is typified by apparently normal development until 6–18 mo of age, when motor and communicative skills regress and hand stereotypies, autonomic symptoms, and seizures present. Restoration of MeCP2 function selectively to astrocytes reversed several deficits in a murine model of RTT, but the mechanism of this rescue is unknown. Astrocytes carry out many essential functions required for normal brain functioning, including extracellular K^+^ buffering. Kir4.1, an inwardly rectifying K^+^ channel, is largely responsible for the channel-mediated K^+^ regulation by astrocytes. Loss-of-function mutations in Kir4.1 in human patients result in a severe neurodevelopmental disorder termed EAST or SESAME syndrome. Here, we evaluated astrocytic Kir4.1 expression in a murine model of Rett syndrome. We demonstrate by chromatin immunoprecipitation analysis that Kir4.1 is a direct molecular target of MeCP2. Astrocytes from *Mecp2*-deficient mice express significantly less Kir4.1 mRNA and protein, which translates into a >50% deficiency in Ba^2+^-sensitive Kir4.1-mediated currents, and impaired extracellular potassium dynamics. By examining astrocytes in isolation, we demonstrate that loss of Kir4.1 is cell autonomous. Assessment through postnatal development revealed that Kir4.1 expression in *Mecp2*-deficient animals never reaches adult, wild-type levels, consistent with a neurodevelopmental disorder. These are the first data implicating a direct MeCP2 molecular target in astrocytes and provide novel mechanistic insight explaining a potential mechanism by which astrocytic dysfunction may contribute to RTT.

## Visual Abstract

**Figure:**
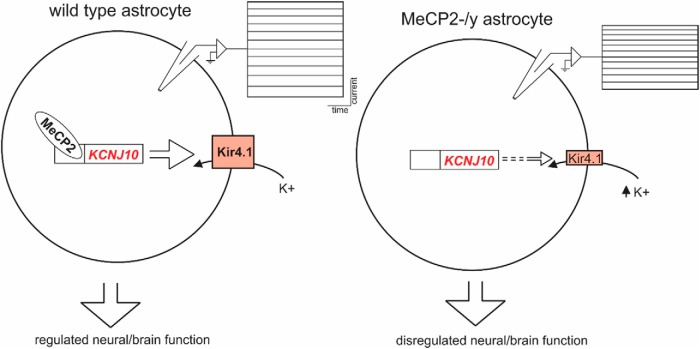

## Significance Statement

Rett syndrome is a devastating neurodevelopmental disorder that affects 1 in 10,000–25,000 females. Mutations in methyl-CpG-binding protein 2 (MeCP2), a transcriptional regulator, are responsible for >95% of RTT cases. Recent work has shown that astrocytes contribute significantly to the disorder, although their contribution to this disease is not known. Here, we demonstrate that the critical astrocyte K^+^ channel Kir4.1 is a novel molecular target of MeCP2. MeCP2 deficiency leads to decreased *Kcnj10*/Kir4.1 mRNA levels, protein expression, and currents. These findings provide novel mechanistic insight and begin to elucidate the role of astrocytes in this disorder.

## Introduction

Rett syndrome (RTT) is an X-linked neurodevelopmental disorder that affects 1 in 10,000–25,000 females (Percy, 2002). RTT is characterized by apparently normal development until 6–18 mo of age, when deficits in speech, ambulation, and hand use (e.g., hand wringing, clapping) become apparent (Neul et al., 2010). Breathing disturbances, scoliosis, diminished pain response, and seizures are also commonly associated with RTT (Jian et al., 2007; Glaze et al., 2010; Neul et al., 2010). More than 95% of girls with RTT have a mutation in the methyl-CpG-binding protein 2 (*MECP2*) gene located on the X-chromosome (Cuddapah et al., 2014). Knockout or mutation of *Mecp2* in mice recapitulates symptoms associated with RTT (Chen et al., 2001; Guy et al., 2001). Functionally, MeCP2 is a methyl-CpG–binding protein, which binds methylated and unmethylated DNA to modulate gene activity, and can act as a repressor or an activator of gene transcription depending on the context (Chahrour et al., 2008).

MeCP2 is most highly expressed in neurons, but it is also expressed in glia cells (Ballas et al., 2009; Kifayathullah et al., 2010; Zachariah et al., 2012; Liu et al., 2015, 2017). In addition, there are indications that astrocytic dysfunction plays a role in the pathophysiology of RTT. RTT astrocytes are abnormal (Maezawa et al., 2009), with altered microtubule assembly (Nectoux et al., 2012) and glutamate clearance (Okabe et al., 2012). Also, when cultured with MeCP2-deficient glia, wild-type (WT) neurons display aberrant morphology (Ballas et al., 2009; Williams et al., 2014). Specific deletion of MeCP2 in astrocytes also resulted in disturbed breathing patterns (Garg et al., 2015). Importantly, postnatal re-expression of MeCP2 in astrocytes in globally *Mecp2*-deficient mice improved locomotion, decreased anxiety, increased lifespan, and normalized respiration, neuronal cell size, and dendritic morphology (Lioy et al., 2011). However, to date, few direct molecular links between astrocytes and astrocytic dysfunction in animal models of RTT have been demonstrated. Possibly shedding some light on this question, a recent transcriptomic study in MeCP2-deficient mice indicates that 46 genes that are unique to astrocytes are disrupted in RTT, one being *Kcnj10*, the gene that codes for Kir4.1 (Pacheco et al., 2017).

*Kcnj10* expression is in the top 1% of all expressed genes in astrocytes (Zheng et al., 2014). Underscoring the relative importance of this channel, knockout or mutation of *KCNJ10* causes seizures, ataxia, and developmental deficits in mice and humans alike (Djukic et al., 2007; Bockenhauer et al., 2009; Scholl et al., 2009). Direct evidence that loss of Kir4.1 alters neuronal function was recently demonstrated in a mouse model of Huntington’s disease, where AAV-mediated restoration of Kir4.1 expression specifically to astrocytes reduced the concentration of extracellular K^+^ ([K^+^]_o_), prolonged survival, and ameliorated motor abnormalities observed in these mice (Tong et al., 2014). Furthermore, a compound heterozygous change (two missense mutations) in *KCNJ10* was reported in a patient with RTT-like symptoms who lacked a mutation in *MECP2* (Sajan et al., 2017).

In the current study, we demonstrate that Kir4.1 is a direct molecular target of MeCP2. *In situ*, astrocytes from *Mecp2*-deficient mice show smaller astrocytic K^+^ currents, with concomitant significant reductions in Kir4.1 protein and transcription. These data suggest MeCP2 is a positive regulator of *Kcnj10* gene expression through development and potentially provide insight explaining how astrocytic dysfunction may contribute to RTT.

## Materials and Methods

### Animals

All animal procedures were performed in accordance with the [Author University] animal care committee’s regulations. Every effort was made to minimize pain and discomfort. WT males were bred with heterozygous *Mecp2^tm1.1Jae^* (Jaenisch mutation) female mice, which lack exon 3 in the *Mecp2* gene (Chen et al., 2001). Genotypes of offspring were confirmed by PCR of DNA isolated from tail clips. Mutant male mice (*Mecp2^–/y^*) were used for experimentation after postnatal day 50 (P50) when symptomatic, as demonstrated by hypoactivity and hindlimb clasping on suspension from tail compared with WT littermates of the same age. Symptomatic mutant female mice (*Mecp2*±) were used for experimentation at 7–8 mo of age and compared with WT female mice of the same age. All animals were on a C57BL/6 background.

### Western blotting

Animals were anesthetized with CO_2_ and quickly decapitated. Brains were removed and placed in ice-cold PBS. Under a binocular microscope, the cerebellum and brainstem were removed, and the cortex was separated from hippocampus and midbrain. Cortex, hippocampus, midbrain, cerebellum, and brainstem were collected. Briefly, tissue was placed in ice-cold homogenization buffer (100 mm Tris, pH 7.5, 1% SDS at 50 mg/ml) supplemented with protease and phosphatase inhibitors (Sigma-Aldrich) and sonicated for 10 s. Tissue homogenates were centrifuged for 5 min at 12,000 × *g* at 4°C. Protein quantification was performed on the supernatant using a DC protein assay kit from Bio-Rad. Protein was heated to 60°C for 15 min in an equal volume of 2× sample buffer (100 mm Tris, pH 6.8, 10% SDS in Laemmli-SDS, and 600 mm β-mercaptoethanol). Equal amounts of protein were loaded into a 4%–20% gradient precast SDS gel (Bio-Rad). Gels were transferred at 100 V for 1 h at room temperature to PDVF membrane (Millipore). Membranes were blocked in blocking buffer (10% dried milk in TBST) for 1 h before probing with antibodies. The blots were probed with rabbit anti-Kir4.1 (Alomone, 1:1000 for 1 h), washed three times in TBST, and probed with an HRP-conjugated secondary antibody (Santa Cruz Biotechnology) for 1 h. After three 10-min washes, membranes were developed with Classico chemiluminescent reagent (Millipore) using a Kodak film developing system (Kodak). The blots were then stripped and reprobed for chicken anti-GAPDH (Abcam, 1:2000 for 1 h) or rabbit anti–β-tubulin (Millipore, 1:5000 for 30 min), which was used as a loading control. Protein expression was quantified using ImageJ. Target protein was normalized to GAPDH expression in the same lane. Both tetrameric (∼150-kDa) and monomeric (∼50-kDa) Kir4.1 isoforms were detected (e.g., Fig. 3*A*) that are specific to Kir4.1, as they are not present in *Kcnj10* knockout animals or negative control lysates (Olsen et al., 2006). The analysis included the entire lane (both isoforms). Cortical and brainstem Western blots were run in triplicate or quadruplicate. Human embryonic kidney (HEK) cell lysates were used as a negative control for astrocytic proteins on Western blots. Relative amounts of protein are reported.

### Quantitative PCR

mRNA was isolated from either cultured astrocytes or cortical and brainstem tissue collected as above using an RNA isolation kit (Qiagen). mRNA was converted to cDNA using the VILO Superscript kit (Life Technologies). Quantitative real-time PCR was run using TaqMan specific probes (all from Life Sciences) for *Kcnj10* (Kir4.1, Mm00445028_m1), Gfap (GFAP, Mm01253033), *Hexb* (hexosaminidase B, Mm01282432_m1), *Tmem119* (transmembrane protein 119, Mm00525305_m1), *Mbp* (myelin basic protein, Mm01266402_m1), *Rbfox3* (NeuN, Mm01248771_m1), and *Gapdh* (GAPDH, Mm99999915_g1). All probes span exon boundaries and as such only amplify mRNA. Each sample was run in triplicate and normalized to *Gapdh*, and the comparative Ct method was used to calculate changes in gene expression.

### Astrocyte isolation

Astrocyte isolation was performed according to Holt and Olsen (2016). Briefly, mice were anesthetized with CO_2_ and rapidly decapitated, and their cortices were microdissected in ice-cold cutting solution (120 mm NaCl, 3 mm KCl, 2 mm MgCl_2_, 0.2 mm CaCl_2_, 26.2 mm NaHCO_3_, 11.1 mm glucose, and 5 mm Hepes), supplemented with AP5 (3 mm) and CNQX (3 mm) and bubbled with 95% oxygen. The tissue was minced and enzymatically dissociated with a Papain Dissociation kit (Worthington) following manufacturer’s instructions. After dissociation, myelin debris and microglia were removed using a Myelin Removal Kit (Miltenyi Biotec) and Cd11b^+^ Microbeads (Miltenyi Biotec), respectively. Astrocytes were then acutely isolated using anti–ACSA-2^+^ (astrocyte cell surface antigen 2) MicroBead kit (Miltenyi Biotec). The ASCA-2^+^ epitope is from the astrocyte-specific β2 subunit of the sodium potassium exchanger (Batiuk et al., 2017). The anti–ACSA-2^+^ antibody is highly specific for astrocytes (Holt and Olsen, 2016), is robustly expressed in astrocytes (Kantzer et al., 2017), and is used as a first choice for isolating astrocytes (Batiuk et al., 2017). Manufacturer instructions were generally followed, except incubation times were extended to 25 min and total volume of microbeads was increased to 20–40 μl.

### Primary astrocyte cultures

Astrocytes from RTT or WT littermates were isolated from P3–P6 pups as described above. Astrocytes were plated on 13-mm glass coverslips coated with poly-l-ornithine and laminin in 24-well plates at a density of 1.0 × 10^5^ cells. Astrocytes were maintained in serum-free media (50% Neurobasal Medium (Thermo Fisher Scientific), 50% MEM, 1 mm sodium pyruvate, 2 mm glutamine, and B27). Medium was changed every day for 3 consecutive days, with subsequent media changes every 3–4 d. RNA was collected after 7 and 14 days *in vitro* (DIV) using Ambion’s PureLink RNA Isolation Kit.

### Primary astrocyte culture immunohistochemistry

Astrocytes were fixed for immunofluorescence after 7 DIV. Cells were first washed with cold PBS, followed by fixation with 4% paraformaldehyde for 15 min at room temperature. Subsequently, cells were incubated for 1 h in blocking buffer (10% goat serum, 0.3% Triton X-100 in PBS). Astrocyte processes were stained with overnight incubation with Dako anti-rabbit GFAP (Z0334) at 1:1000 concentration. Fluorescent images were acquired with an Olympus VS-120 system.

### Acute brain slice preparation

Mice were anesthetized with CO_2_ and rapidly decapitated. Brain was quickly isolated and placed in icy artificial cerebrospinal fluid (ACSF) cutting solution (see above). All solutions were continuously bubbled with 95% O_2_/5% CO_2_ to bring to pH 7.4. Coronal 300-μm slices were cut with a Leica VT1000A vibratome and placed in ACSF at room temperature containing the following in mm: 120 NaCl, 3 KCl, 1 MgCl_2_, 2 CaCl_2_, 26.2 NaHCO_3_, 11.1 glucose, and 5 Hepes. Slices were allowed to recover for 45 min at room temperature before use.

### K^+^-sensitive microelectrodes

Borosilicate glass capillary tubes (Warner Instrument Corp.; GC200F-10; OD: 2.0 mm, ID: 1.16 mm) were washed in concentrated nitric acid, water, then 100% EtOH and dried overnight at 200°C. Pipettes were pulled on Sutter Instrument Model P-97 and then placed upright and incubated above a dish containing silane [bis(dimethylamino)dimethylsilane] for 5 min at 200°C. Using a fine-tipped syringe (Microfil 28 AWG, World Precision Instruments), a K^+^ ion exchanger (IE190, World Precision Instruments) was filled at the pipette tip, with care taken to ensure no bubbles. Pipettes were then backfilled with 100 mm KCl. K^+^-sensitive microelectrodes were calibrated with solutions containing in mm: 133 NaCl, 3 KCl, 1.5 CaCl_2_, 1.2 NaH_2_PO_4_, 1 MgCl_2_, 10 glucose, and 8.55 Hepes or 106 NaCl, 30 KCl, 1.5 CaCl_2_, 1.2 NaH_2_PO_4_, 1 MgCl_2_, 10 glucose, and 8.55 Hepes after every experiment. All experiments were performed using a World Precision Instruments electrometer (model FD 223), digitized with a Digidata 1440A (Molecular Devices), and stored and analyzed using Clampex 10.2. K^+^ responses were recorded, at depths 40–75 μm into the slice, in layer II/III of the cortex resulting from 3 consecutive 1-s stimulations of 100 μA at 20 Hz (0.1-μs pulse width) in layer IV/V of the cortex. Signals were low-pass filtered at 10 Hz.

### Whole-cell patch clamp electrophysiology

Patch pipettes were pulled from thin-walled borosilicate glass (World Precision Instruments, catalog no. TW150F-4) and filled with pipette solution containing the following in mm: 125 K-gluconate, 10 KCl, 10 Hepes (free acid), 10 disodium creatine phosphate, 2 MgATP, 0.2 NaGTP, and 0.5 EGTA. The pipette solution was brought to pH 7.3 with KOH and 285–290 mOsm with sucrose. After filling patch pipettes with pipette solution, final resistances were 6–8 MΩ. Slices were transferred to a Zeiss Examiner D1 equipped with a 40× water-immersion lens and Zeiss Axiocam MRm for imaging and constantly perfused with ∼30°C ACSF. Astrocytes were identified using morphologic features. Recordings were made with an Axopatch 200B amplifier (Molecular Devices), low-pass filtered at 1 kHz, and digitized with Digidata 1440A (Molecular Devices). Data were acquired and stored on a personal computer using Clampex 10.2. Cell capacitance and series resistance were compensated for using the amplifier. Cell capacitance was measured from the amplifier. Experiments with a series resistance up to 12 MΩ were used, and series resistance was compensated for by 80% to reduce voltage errors. Steady-state currents were measured.

### Chromatin immunoprecipitation

Half-brains from 7–9-wk-old *Mecp2^+/y^* and *Mecp2^–/y^* mice were collected for chromatin immunoprecipitation (ChIP) analysis. Chromatin preparation was performed using the Magna ChIP G Tissue Kit (Millipore) according to manufacturer’s instructions. Briefly, samples were sheared in 1× Tissue Stabilizing Solution (Millipore) with protease inhibitors using a 1-ml pipette tip. Samples were then fixed in 1% formaldehyde for 10 min at 37°C with minor agitation. The fixation was quenched using 10× glycine (for final concentration at 1×) and incubated for 5 min at room temperature. Cross-linked samples were washed 3 times in ice-cold 1× PBS with protease inhibitors. Samples were then lysed on ice in Tissue Lysis Buffer with protease inhibitors for 15 min, with brief vortexing every 5 min. Samples were resuspended in 600 µl ChIP Dilution Buffer with protease inhibitors and sonicated on wet ice under the following conditions: 10 s pulse, 50 s rest, 20% amplitude, 5 total cycles. After sonication, samples were centrifuged at 15,000 rcf at 4°C for 10 min. The resulting supernatant was collected and distributed into 200-µl aliquots for immunoprecipitation (IP).

IP, washes, reverse cross-linking, and DNA collection and purification were performed as outlined in Li et al. (2012) with the following modifications. (1) Additional ChIP Dilution Buffer with protease inhibitors was added to each aliquot for a final volume of 500 µl. (2) Both IPs and inputs were digested using 10 µl of 0.5 m EDTA, 20 µl of 1 m Tris, pH 6.5, and 1 µl of 20 mg/ml proteinase K (Clontech) for 1 h at 55°C with minor agitation. DNA was collected and purified using the Qiagen PCR Purification kit according to manufacturer’s instructions with the following modifications. (1) At the final elution step, EB buffer was incubated on the column for 10 min before centrifugation. 2) Final DNA was eluted in 50 µl. Quantitative PCR (qPCR) was performed on the Bio-Rad CFX96 machine using the Applied Biosystems SYBR Select Master Mix for CFX. qPCR cycling parameters were as follows: 95°C for 3 min, followed by 40 cycles of 95°C for 10 s, 60°C for 30 s, and 72°C for 45 s, and a final incubation at 70°C for 10 min. Primer efficiency was verified through melting-curve analysis (60°C for 1 min, and 0.5°C increments starting at 60°C up to 95°C for 15 s each) and running qPCR products on a 1.5% agarose gel. Primers used to amplify specific regions within *Kcnj10* promoter region are listed in Table 1. The ddCT method was used to determine fold change expression in *Mecp2^+/y^* relative to *Mecp2^–/y^* mice. Briefly, the ddCT was calculated as the following:

$$\text{dCT}={\text{CT}}^{\text{IP}\left(\text{MeCP2 or IgG}\right)}–\left({\text{CT}}^{\text{Input}}-\text{6}.\text{644}\right),$$

where 6.644 represents 1% starting input;

$$\text{ddCT}=\text{dCT}\left(Mecp2^{+/y}\text{or }Mecp2^{-/y}\right)–{\text{average MeCP2}}^{-/\text{Y}}\text{dCT}.$$

Fold change expression was calculated using the formula 2^–ddCT^ for both *Mecp2^+/y^* and *Mecp2^–/y^* mice.

**Table 1.** Targeted Kir4.1 CpG island I (promoter) PCR amplification sites

Target name	Forward primer sequence	Reverse primer sequence
CpG I-1	5′-AGTTTCCCTGCTTTCAATCCTG-3′	5′-CCTGTGGGAACACAGACACA-3′
CpG I-2	5′-GGATGGGAAGAGTTTGACGC-3′	5′-TACGGTGCAAAGTGTGGGAG-3′
CpG I-3	5′-CACACTTTGCACCGTACTGC-3′	5′-GATAGAAGCCGAGCTGGCAA-3′
CpG I-4	5′-GGCCGCCTCACTTTTCTTCT-3′	5′-TGGAGAGATTTGGGCAAGGC-3′

### Data analysis

Data were organized in Microsoft Excel or Origin 8.5. Unless stated otherwise, two-tailed *t* tests were performed as appropriate, except for non–normally distributed data, in which case Mann–Whitney nonparametric test was performed. In Fig. 5, two-way ANOVA was performed with Tukey *post hoc* comparison. The statistical tests were performed using InStat 3 (GraphPad Software) or MYSTAT (Systat Software). All data are reported as average ± SEM.

## Results

### Kir4.1 currents are significantly decreased in Mecp2^–/y^ mice

Astrocytes contribute to symptomology of RTT (Lioy et al., 2011). However, few studies have examined the functional differences between WT and RTT astrocytes. Kir4.1, a glia-specific inward rectifying channel, is critical for normal CNS function. Mutations in *KCNJ10* have been linked to developmental disorders characterized by early onset seizures, ataxia, epilepsy, and profound developmental delay (Kofuji et al., 2000). Of note, these symptoms are also observed in RTT. We therefore sought to investigate the properties of Kir4.1 channel function and expression in RTT.

**Figure 1.**
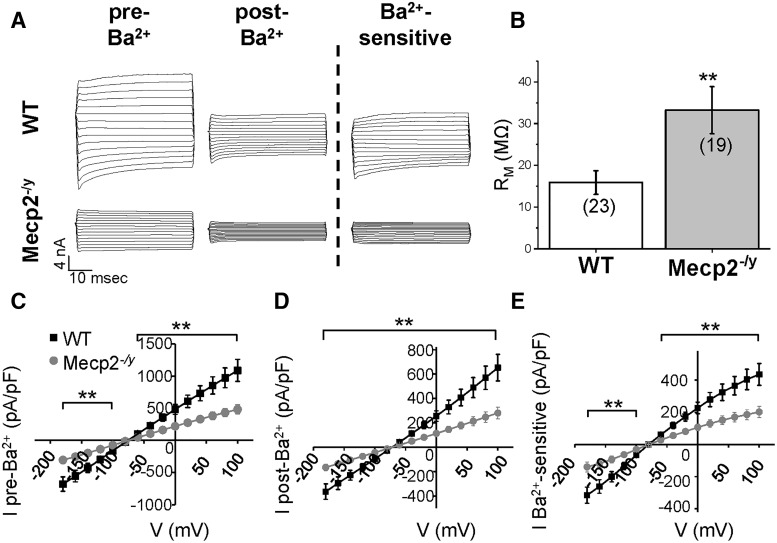
Ba^2+^-sensitive Kir4.1 currents are significantly reduced in layer II/III astrocytes of *Mecp2^–/y^* mice. ***A***, Representative traces of pre-Ba^2+^, post-Ba^2+^, and Ba^2+^-sensitive currents in WT and *Mecp2^–/y^* astrocytes. ***B***, *Mecp2^–/y^* astrocytes show higher input resistance than their WT littermates. ***C–E***, Current–voltage graphs (*n* = 19–24) demonstrate that pre-Ba^2+^, post-Ba^2+^, and Ba^2+^-sensitive currents are significantly reduced in *Mecp2^–/y^* astrocytes [except at the –80-mV step in the pre-Ba^2+^ currents (***C***) and the Ba^2+^-sensitive currents (***E***)]. In all, results are consistent with a reduction of Kir4.1 currents in the plasma membrane. Mann–Whitney test was conducted for each voltage step between the two genotypes. **, *p* < 0.01.

MeCP2 expression is high in cortical tissue and apparent cortical dysfunction is observed in *Mecp2*-deficient mice (Kishi and Macklis, 2004; D’Cruz et al., 2010). Kir4.1 currents were measured in layer I and layer II/III cortical astrocytes of symptomatic *Mecp2* mutant male (*Mecp2^–/y^*) mice and littermate age- and sex-matched controls. To isolate Kir4.1 currents, we stepped cortical astrocytes from a holding potential of –80 to 0 mV, and then from –180 to 100 mV in 20-mV increments. We washed on 100 μM BaCl_2_, a concentration that specifically blocks Kir channels (Ransom and Sontheimer, 1995), and subtracted out currents that were sensitive to BaCl_2_. As there was no significant difference between astrocytes in layer I and layer II/III (data not shown), we pooled both layers together. Astrocyte recordings obtained from WT mice (*n* = 24) displayed large-amplitude, linear currents typical of passive astrocytes (Zhou et al., 2006). As depicted in Fig. 1*E*, the “passive” Ba^2+^-sensitive Kir4.1 currents were smaller in *Mecp2^–/y^* astrocytes (*n* = 19). Although whole-cell currents were significantly smaller in *Mecp2^–/y^* astrocytes (Fig. 1*C*), which could be partially explained by smaller Ba^2+^-sensitive Kir4.1 currents, there was also a decrease in other unidentified Ba^2+^-insensitive currents in *Mecp2^–/y^* astrocytes (Fig. 1*D*). In an attempt to identify other K^+^ channels disrupted in the cortex of MeCP2-deficient mice, we mined RNA sequencing data from a recent transcriptomic study performed in the cortex of WT and symptomatic MeCP2-deficient mice that were age matched with the current study. Of the 15 *Kcnj* genes identified in that RNA sequencing study, only *Kcnj10* (Kir4.1) and *Kcnj16* (Kir5.1, a channel thought to form heteromers with Kir4.1, but not homomeric channels) were significantly downregulated in the cortex of MeCP2-deficient mice (Table 2). Of the 14 *Kcnk* genes identified, only *Kcnk2* (Trek-1) was differentially expressed, and it was modestly upregulated. Together, these data suggest that a loss of Kir4.1 homomers and possibly Kir4.1/Kir5.1 heteromeric channels contribute to the reduced K^+^ conductance observed in MeCP2-deficient mice.

**Table 2.** RNA sequencing indicates 14 *kcnj* and 15 *kcnk* potassium channels identified in the cortex of symptomatic MeCP2^^–/y^^ mice and WT age-matched littermates

Protein	Gene name	FPKM WT	FPKM TG	Fold change	*q* value	Cell type specificity
Kir4.1	*Kcnj10*	80.991	65.281	–0.311	0.00065	Astrocytes/oligodendrocytes/OPCs
Kir5.1	*Kcnj16*	14.832	11.353	–0.386	0.00853	Astrocytes/OPCs
TREK-1	*Kcnk2*	25.299	31.559	0.319	0.03503	Astrocytes/neurons/OPCs

Only *Kcnj10*, *Kcnj16* and *Kcnk2* are differentially expressed (data mined from Pacheco et al., 2017).

Kir4.1 channels contribute to the intrinsic membrane properties of astrocytes (Kofuji et al., 2000; D’Ambrosio et al., 2002; Neusch et al., 2006; Olsen et al., 2006; Djukic et al., 2007; Chever et al., 2010; Ma et al., 2014). Therefore, we examined input resistance and resting membrane potential (RMP) in WT and *Mecp2^–/y^* cortical astrocytes. Kir4.1 has a high open probability at rest (Nwaobi et al., 2016), and the relative expression levels of Kir4.1 correlate with the astrocyte input resistance. Lower Kir4.1 channel activity in *Mecp2^–/y^* mice was associated with significantly higher membrane resistance (Fig. 1*B*; 15.9 ± 2.8 MΩ in WT vs. 33.2 ± 5.6 MΩ in *Mecp2^–/y^*; *p* = 0.006; *n* = 19–23). However, both genotypes showed similar hyperpolarized resting membrane potential (–77.9 ± 0.7 mV in WT vs. –76.6 ± 0.9 mV in *Mecp2^–/y^*; *p* = 0.23; *n* = 19–24) and whole-cell capacitance, a measure of cell size (18.5 ± 1.5 pF in WT vs. 20.3 ± 2.9 pF in *Mecp2^–/y^*; *p* = 0.56; *n* = 19–24). The lack of a change in RMP is in contrast to previous reports regarding the importance of Kir4.1 to the RMP of astrocytes (Olsen et al., 2006; Djukic et al., 2007; Seifert et al., 2009). The lack of a change in resting membrane potential in RTT astrocytes may be explained by the partial reduction in current in *Mecp2^–/y^* mice as opposed to complete knockout (Kir4.1 KO mice) or knockdown (siRNA knockdown in cultured astrocytes) as published in previous reports. Astrocytes are thought to be selectively permeable to K^+^ ions; as such, the remaining Kir4.1 and leak channels would maintain the resting membrane potential near the K^+^ equilibrium potential. Alternatively, the increased *Kcnk2* gene expression in the cortex of MeCP2^–/y^ mice may compensate for lower levels of Kir4.1 expression.

### [K^+^]_o_ homeostasis is dysregulated in MeCP2^–/y^ mice

**Figure 2.**
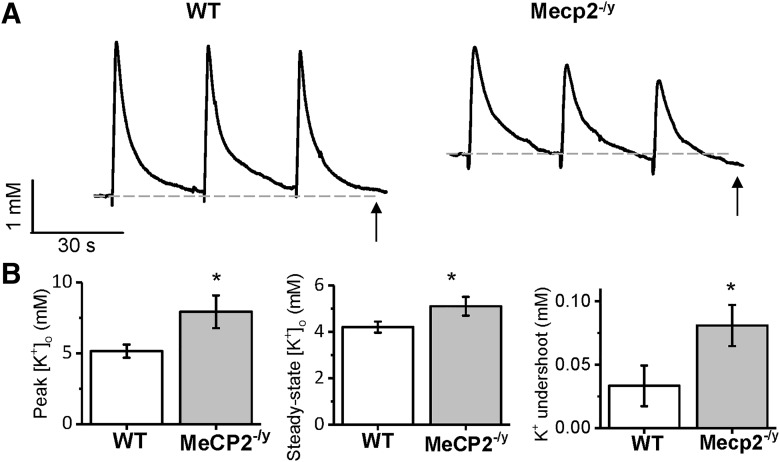
[K^+^]_o_ is elevated and [K^+^]_o_ undershoots after stimulation in MeCP2^–/y^ cortex. ***A***, Representative changes in [K^+^]_o_ after three successive stimulations in WT and *Mecp2^–/y^* slices. Baseline [K^+^]_o_ (dashed horizontal line) is elevated in *Mecp2^–/y^* slices. ***B***, Left, peak [K^+^]_o_ increase after insertion of K^+^-sensitive microelectrode into slice is greater in *Mecp2^–/y^* slices. Middle, steady-state [K^+^]_o_ is elevated in *Mecp2^–/y^* slices. Right, K^+^ undershoot after the second stimulation is greater in *Mecp2^–/y^* slices. *, *p* < 0.05.

Given the previously demonstrated role of astrocytes in the buffering and maintenance of [K^+^]_o_ (Kofuji and Newman, 2004), we assessed whether K^+^ homeostasis is disrupted in *Mecp2^–/y^* mice. To test this, we stimulated neuronal activity in layer IV/V of the cortex with a 1-s stimulus (20 Hz, 100 μA) followed by a 30-s recovery, performed 3 times in series. Changes in [K^+^]_o_ were measured in layer II/III with a calibrated K^+^-sensitive microelectrode. Upon insertion of the K^+^-sensitive microelectrode into the slice, we detected a spike in [K^+^]_o_ followed by a fall to a steady-state level. Consistent with the possibly that MeCP2 regulates Kir4.1 expression and consequentially K^+^ homeostasis, we found the peak of this K^+^ spike to be larger in *Mecp2^–/y^* mice compared with WT littermates (5.2 ± 0.5 mm in WT vs. 7.9 ± 1.2 in *Mecp2^–/y^*; Fig. 2*B*, left; *p* < 0.05; *n* = 7–8). Additionally, steady-state [K^+^]_o_ was higher in *Mecp2^–/y^* mice compared with WT littermates (4.2 ± 0.2 mm in WT vs. 5.1 ± 0.4 in *Mecp2^–/y^*; Fig. 2*A*, dashed line, Fig. 2*B*, middle; *p* < 0.05; *n* = 7–8). Intriguingly, a similar elevation in baseline K^+^ was observed in a murine model of Huntington disease, which showed similar reductions in astrocyte Kir4.1-mediated currents (Tong et al., 2014). We also observed an increase in the amplitude of the K^+^ undershoot after the second stimulation (Fig. 2*A*, arrows). The amplitude of the undershoot under baseline increased from 0.03 ± 0.02 mm in WT to 0.08 ± 0.02 mm in MeCP2^–/y^ mice (Fig. 2*B*, right; *p* < 0.05; *n* = 6–9). Using a similar approach, Chever et al. (2010) demonstrated the appearance of a significant undershoot after stimulation in the hippocampus of GFAP-targeted Kir4.1 knockout mice. Together, these data indicate that K^+^ homeostasis is disrupted in MeCP2^–/y^ mice and support the possibility that loss of MeCP2 disrupts Kir4.1 function.

### Kir4.1 protein expression is significantly downregulated in Mecp2^–/y^ mice

**Figure 3.**
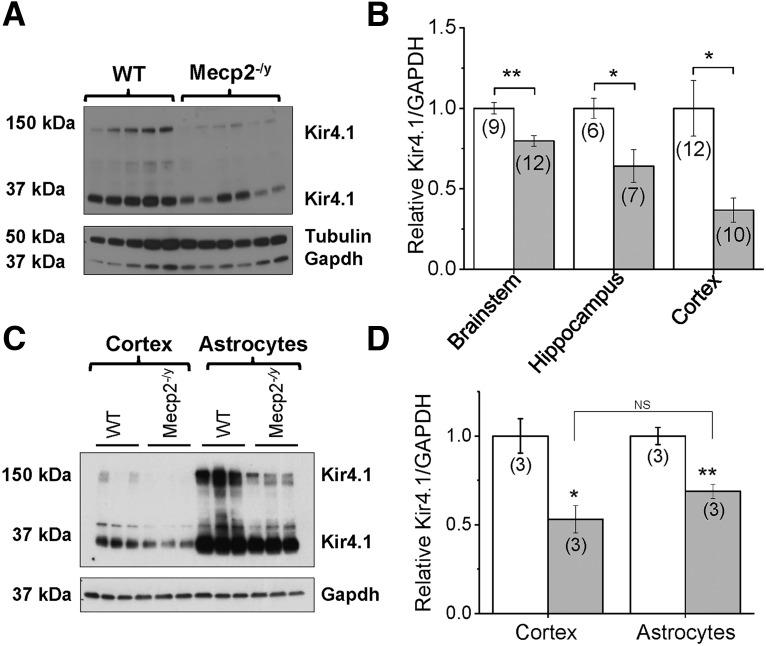
Kir4.1 protein is downregulated in *Mecp2^–/y^* brains. ***A***, Representative Western blot of cortical protein lysates from 5 wild-type and 6 symptomatic *Mecp2^–/y^* males demonstrates a significant loss of Kir4.1 protein expression in cortex (∼150 kDa tetramer, 37 kDa monomer) when normalized to a loading control (GAPDH or tubulin). ***B***, Quantification of ImageJ analysis normalizing Kir4.1 protein expression to GAPDH in cortex. Similar loss of Kir4.1 immunoreactivity in *Mecp2*-deficient male mice is seen in brainstem and hippocampus. In isolated astrocytes, Kir4.1 expression is lower in *Mecp2^–/y^* mice compared to WT, as in whole-cortex lysate (***C***, ***D***).

To investigate whether decrease in currents stems from decreased Kir4.1 protein expression, we examined Kir4.1 expression in the cortex. We isolated the cortex through microdissection, and Western blot analysis revealed 63% downregulation of Kir4.1 protein expression in *Mecp2^–/y^* mice compared with littermate controls, with GAPDH and tubulin used as loading controls (Fig. 3*A*, *B*; *p* < 0.05; *n* = 10–12). The bands on the Western blot represent both tetrameric (∼150-kDa) and monomeric (∼50-kDa) isoforms of Kir4.1 (Fig. 3*A*), which are commonly observed when blotting for Kir4.1 and are not observed in negative control samples (HEK cells or Kir4.1 KO mice; Olsen et al., 2006). Intriguingly, higher multimers of Kir4.1 are associated with developmental upregulation of the channel that occurs during postnatal development and astrocytic maturation (Dibaj et al., 2007), and these higher molecular weight bands are reduced in MeCP2-deficient mice.

We have recently shown that Kir4.1 is expressed at significantly higher levels in the brainstem relative to other CNS structures (Nwaobi et al., 2014). Therefore, we also examined whole-brainstem homogenates from WT and *Mecp2^–/y^* to determine whether Kir4.1 was affected in this structure. Western blots indicate a 20% downregulation of Kir4.1 in the brainstem (Fig. 3*B*; *p* < 0.05; *n* = 9–12). Given that a deficiency of Kir4.1 may also contribute to hyperexcitability in the hippocampus (Calfa et al., 2011b), we probed for Kir4.1 expression and found a 36% downregulation (Fig. 3*B*; *p* < 0.05; *n* = 6–7). Loss of Kir4.1 in cortex, brainstem, and hippocampus may contribute to altered function in these brain regions in *Mecp2^–/y^* mice.

Kir4.1 is most highly expressed in astrocytes but is also expressed in oligodendrocytes and NG2^+^ glia (Nwaobi et al., 2016). To evaluate Kir4.1 expression in WT and *Mecp2^–/y^* in astrocytes specifically, versus a whole-cortical homogenate, magnetic cell separation (MACS) was used (Holt and Olsen, 2016). Astrocytes were isolated from symptomatic MeCP2-deficient males and their age-matched littermates. Western blot analysis from isolated astrocytes in WT and Mecp2-deficient mice indicated that Kir4.1 is significantly decreased (Fig. 3*C*, *D*; *p* < 0.01). The reduction is similar in both whole cortex and isolated astrocytes (Fig. 3*D*).

### Kir4.1 (Kcnj10) RNA expression is significantly downregulated in Mecp2^–/y^ and Mecp2^–/+^ mice

**Figure 4.**
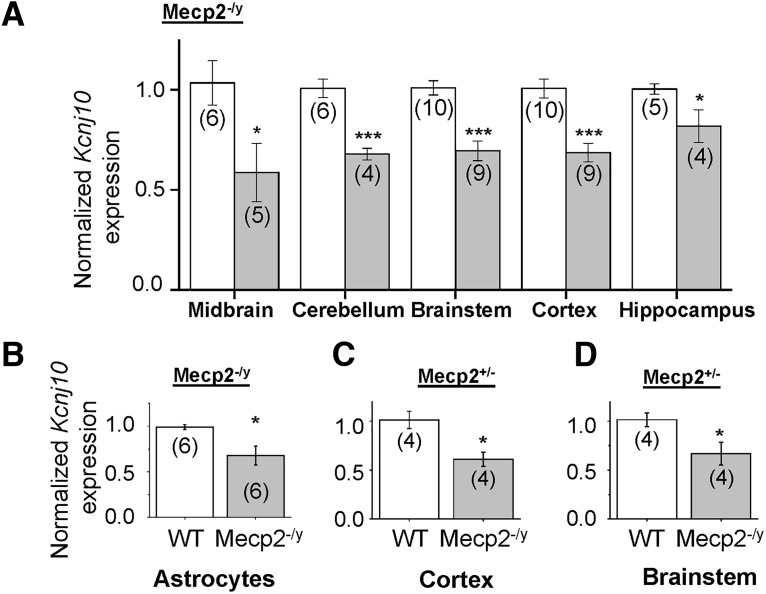
Decreased Kir4.1 transcription in *Mecp2*-deficient mice. ***A***, *Kcnj10* mRNA is significantly downregulated in microdissected cerebellum, midbrain, cortex, hippocampus, and brainstem of *Mecp2^–/y^* mice. ***B***, Similar reduction is observed in isolated cortical astrocytes from *Mecp2^–/y^* mice. ***C***, ***D***, *Kcnj10* is significantly downregulated in the cortex and brainstem of *Mecp2*± symptomatic female mice. *, *p* < 0.05; **, *p* < 0.01; ***, *p* < 0.001.

As we demonstrated a global decrease in expression of Kir4.1 protein in *Mecp2*-deficient mice (Fig. 3), we next examined whether *Kcnj10* transcription is altered in the RTT brain. As a first step, we performed quantitative PCR (qPCR) on microdissected samples isolated from symptomatic *Mecp2^–/y^* mice and WT littermates to examine *Kcnj10* gene expression. In all brain regions, there was a significant loss of *Kcnj10* transcription (Fig. 4*A*): midbrain (41 ± 15%, *p* < 0.05) cerebellum (32 ± 3%, *p* < 0.001), brainstem (31 ± 5%, *p* < 0.001), cortex (31 ± 5%, *p* < 0.001), and hippocampus (18 ± 8%, *p* < 0.05). Additionally, MACS-isolated astrocytes from P60 symptomatic *Mecp2^–/y^* mice cortex also showed reduction in *Kcnj10* transcription, same as in the whole brain (Fig. 4*B*, 33 ± 25%, *p* < 0.05).

Most research involving murine models of RTT involve mutant male mice, although RTT typically affects females. Female mice present a slightly less severe and more heterogeneous symptomology that has a delayed onset, due to variable X-chromosome inactivation (Calfa et al., 2011a). The mutation in our mice, the Jaenisch mutation, expresses in half of the neurons, except at 9-mo-old females in the dentate gyrus, which tend to express less (∼40%) of the chromosome with the mutation (Smrt et al., 2011). Nonetheless, there is no data concerning the effect of X-linked inactivation in glial cells. However, because RTT typically affects females, we asked whether loss of MeCP2 function is also associated with decreased *Kcnj10* mRNA expression in *Mecp2*^±^ mice. Symptomatic *Mecp2*^±^ mice (7–8 mo of age), which displayed motor abnormalities including hindlimb clasping, were used for these studies. We observed a significant decrease in *Kcnj10* gene expression in *Mecp2*^±^ symptomatic females in the cortex (Fig. 4*C*, 1.01 ± 0.08 WT vs. 0.61 ± 0.07 *Mecp2*^±^) and brainstem (Fig. 4*D*, 1.01 ± .07 WT vs. 0.67 ± 0.11 MeCP2^±^). Interestingly, the decrease in mRNA in female *Mecp2*^±^ mice was significantly larger than that seen in males, although females presumably maintain ∼50% MeCP2 expression. The decrease in *Kcnj10* mRNA expression suggests a transcriptional mechanism of regulation.

### MeCP2 regulates Kcnj10 transcription in a cell-autonomous mechanism

**Figure 5.**
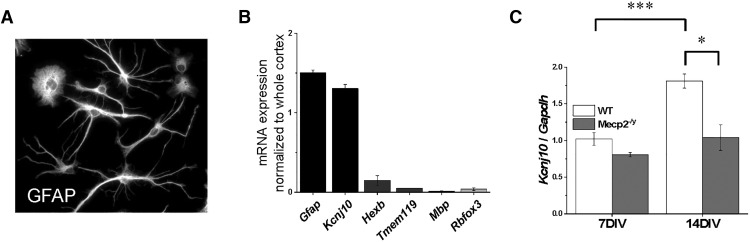
Loss of *Kcnj10* is autonomous to astrocytes. With a magnetic-bead sorting technique, isolated astrocytes were plated and kept in serum-free medium for 7–14 d. Four to six cultures were used, with two biological replicates per culture, a total of 8–12 samples. ***A***, By 7 d, WT astrocytes attain a stellate morphology as indicated by GFAP staining. ***B***, By 14 d in culture astrocytes, *Gfap* and *Kcnj10* expression is enriched compared to age-matched cortex. Cultures express low levels of mRNA for other cell types including microglia (HexB, Tmem119), oligodendrocytes (Mbp), and neurons (Rbfox3), indicating relative purity of the astrocyte cultures. ***C***, When examining the transcription of *Kcnj10*, there is a significant effect for both genotype (*p* < 0.001) and DIV (*p* < 0.001). There was no difference at 7 DIV between WT and *Mecp2^–/y^* astrocytes in transcription of *Kcnj10*. Transcription of *Kcnj10* shows a typical developmental increase in WT astrocytes at 14 DIV, while such an increase was not seen with the *Mecp2^–/y^* astrocytes. Two-way ANOVA was performed with Tukey *post hoc* comparison. *, *p* < 0.05; ***, *p* < 0.001.

The question remains whether MeCP2 controls *Kcnj10* expression via a cell-autonomous mechanism (i.e., direct effect of MeCP2 in astrocytes) or via an indirect effect. To attempt to discern between the two possibilities, we used neuronal-free primary cultured astrocytes. Cortical astrocytes from either WT or *Mecp2^–/y^* P3 mice pups (*n* = 8–12 samples, with 4–6 total cultures, 2 biological replicates per culture) were isolated using the MACS astrocyte isolation as described previously. These astrocytes were cultured for either 7 or 14 DIV, when cells were collected and RNA transcription was measured using qPCR. The cultured astrocytes retained their shaped as assessed by GFAP staining (Fig. 5*A*), and the RNA expression profile fitted that of isolated astrocytes: *Gfap* and *Kcnj10* expression matching whole-cortex expression, whereas markers for other cell types were depleted (Fig. 5*B*). We observed no difference in *Kcnj10* transcription between the two genotypes at 7 DIV (Fig. 5, *p* = 0.98), when *Kcnj10* transcription levels *in situ* are also relatively low. In contrast, *Kcnj10* transcription increases significantly in WT astrocytes after 14 DIV, indicative of normal developmental upregulation (Fig. 5, *p* < 0.001). Transcription in *Mecp2^–/y^* astrocytes did not increase (*p* = 0.99) and was significantly reduced compared with WT astrocytes (*p* < 0.01). These data suggest that loss of *Kcnj10* transcription is autonomous to the mutant astrocyte and results from loss of astrocytic *MeCP2*.

### Kir4.1 is a direct molecular target of MeCP2

**Figure 6.**
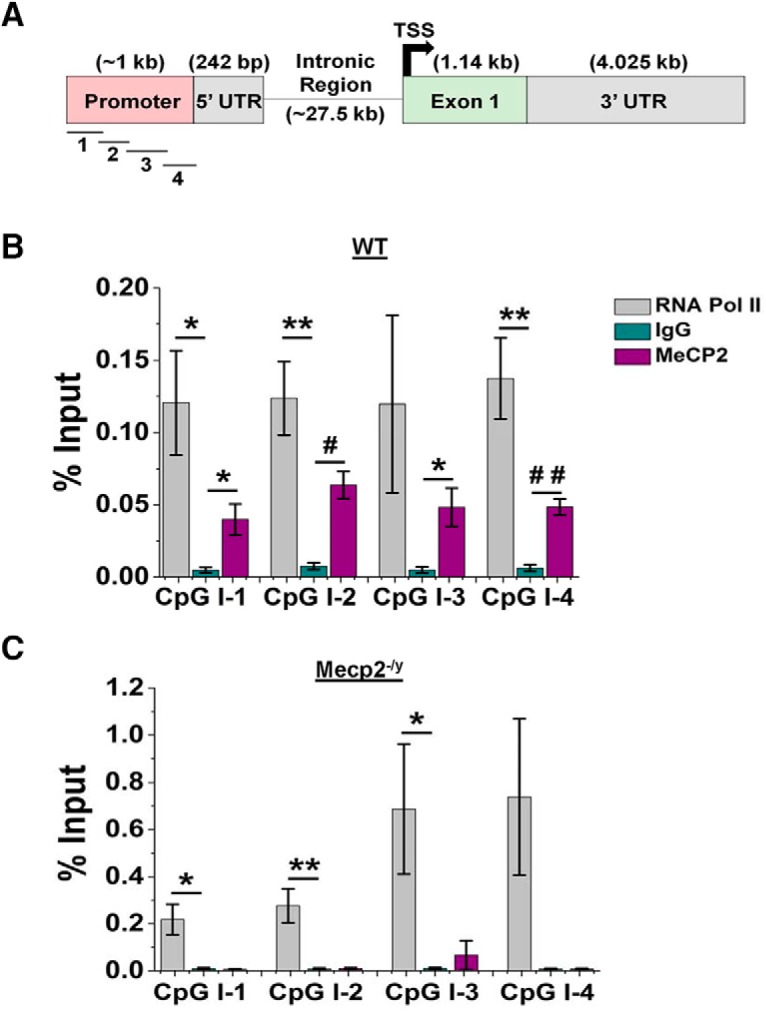
*Kcnj10* is a direct molecular target of MeCP2. ***A***, A schematic of the mouse *Kcnj10* gene indicating the promoter, 5′ UTR, intronic, and exonic region of the gene. The relative locations of primers used to query MeCP2 binding are indicated. ***B***, ***C***, ChIP results indicate significant physical interaction between MeCP2 and *Kcnj10* in three sites in the promoter of the *Kcnj10* gene (***B***, sites 1, 2, and 4, *p* < 0.05, *n* = 5), while no interactions are found in the *Mecp2*-null mice (***C***). *, *p* < 0.05; **, *p* < 0.01; #, *p* < 0.0005; ##, *p* < 0.0001.

Previous work has demonstrated that *Kcnj10* gene expression is robustly, developmentally up-regulated and dependent on the degree of methylation at its promoter (Nwaobi et al., 2014). We postulated that decreased *Kcnj10* transcription might be due to a loss of interaction between MeCP2 and the *Kcnj10* gene in *Mecp2*-deficient mice. To test this, we performed a chromatin immunoprecipitation (ChIP) assay. MeCP2 binds DNA in cytosine-phosphodiester-guanine (CpG) islands that are either methylated or unmethylated to modulate gene expression (Chahrour et al., 2008). To determine whether the entire *Kcnj10* gene contained CpG islands, we performed an *in silico* analysis of mouse *Kcnj10* gene (Fig. 6*A*). Two CpG islands were identified: the first spanned the promoter region and the 5′ UTR, and the second spanned the transcriptional start site and the initial segment of the exonic region. Because MeCP2 is often bound to actively transcribed promoters (Yasui et al., 2007), we designed four (100–150-bp) overlapping primers to amplify the promoter of *Kcnj10* (Table 1). The ChIP analysis was performed by probing for a MeCP2 protein interaction with regions 1–4 in the *Kcnj10* promoter. For these experiments, IgG served as a negative control, and RNA polymerase II (RNA Pol II) served as a positive control. Results from these experiments show significant interactions between MeCP2 and sites 1, 2, and 4 of *Kcnj10* in WT mice (Fig. 6*B*). To confirm the specificity of the immunoprecipitation, we performed the ChIP assay with *Mecp2^–/y^* and did not detect any amplification (Fig. 6*C*). These data are the first demonstrating a direct molecular interaction between MeCP2 and an astrocyte gene target, providing possible mechanistic support for the loss of *Kcnj10* mRNA and its subsequent protein product in *Mecp2*-deficient mice.

### Kir4.1 gene and protein expression are downregulated early in disease progression in MeCP2^–/y^ mice

**Figure 7.**
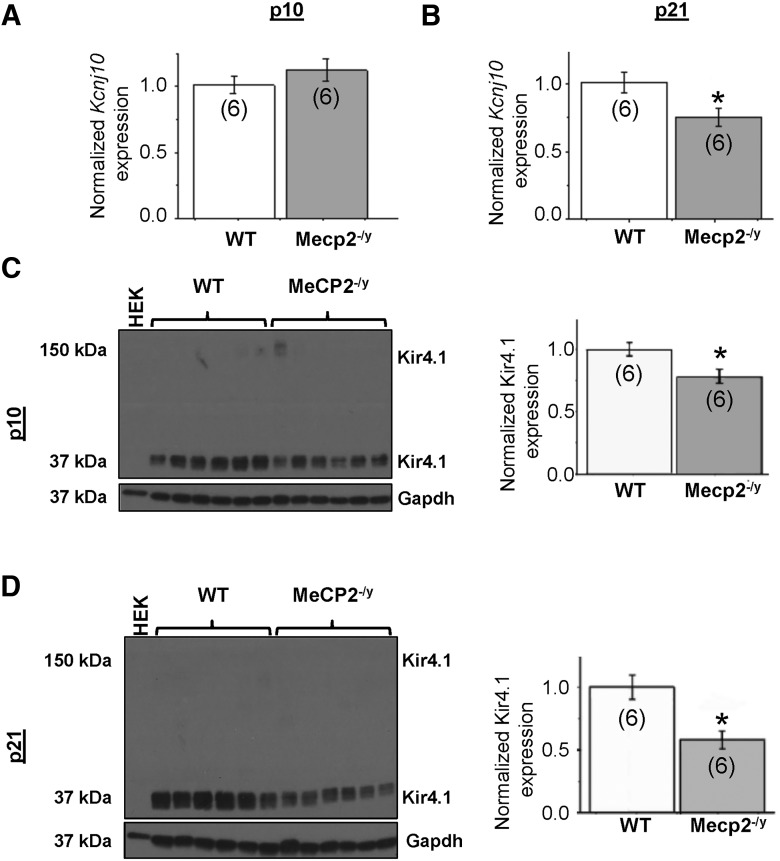
Kir4.1 is not sufficiently upregulated through development in *Mecp2*-deficient mice. ***A***, *Kcnj10* mRNA is not significantly different in the cortex of p10 *Mecp2^–/y^* mice. ***B***, *Kcnj10* mRNA is significantly decreased in the cortex of p21 *Mecp2^–/y^* mice. ***C***, ***D***, Representative Western blots and ImageJ quantification demonstrate loss of Kir4.1 protein expression in cortex in both p10 (***C***) and p21 (***D***) mice when normalized to a loading control (GAPDH). *, *p* < 0.05.

Kir4.1 protein levels are markedly increased during early postnatal development, correlating with increased gene transcription and reduced methylation levels of the *Kcnj10* gene (Nwaobi et al., 2014). The most significant increases in gene and protein expression occur from in postnatal wk 2–4 (Nwaobi et al., 2014), when astrocytes undergo significant morphological refinement and maturation (Bushong et al., 2004). To determine whether MeCP2 deficiency altered normal developmental patterns of *Kcnj10* gene expression, we performed qPCR at two developmental time points (P10 and P21), which are early in the disease progression in RTT mice. No difference in *Kcnj10* transcription was observed at P10 (Fig. 7*A*), but we observed a ∼30% decrease in transcription in the *Mecp2^–/y^* mice by P21 (Fig. 7*B*; *p* < 0.05; *n* = 6). Western blot analysis indicates a significant deficit in Kir4.1 protein levels in *Mecp2^–/y^* (Fig. 7*C*, *D*; *p* < 0.05; *n* = 6) at both time points. These data demonstrate that normal protein levels of Kir4.1 are not achieved at any time point in *Mecp2*-deficient animals, which may be the result of a direct positive interaction between MeCP2 protein and *Kcnj10* gene promoter that is absent in MeCP2^–/y^ mice.

## Discussion

Several recent reports have implicated a role of glia in the progression of RTT (Ballas et al., 2009; Maezawa and Jin, 2010; Lioy et al., 2011; Derecki et al., 2012). Mechanistically, it was hypothesized that RTT astrocytes inhibited neuronal maturation through release of a molecule that stunted dendritic maturity or through deficient release of pro-growth soluble molecule. Previous *in vitro* work also suggests that excess glutamate contributes to abnormal neuronal morphology (Maezawa and Jin, 2010). In the current study, we demonstrate reduced Kir4.1 channel activity in cortical astrocytes. This decrease in channel activity is associated with significant reductions in Kir4.1 protein and mRNA levels and may result from the loss of a direct positive interaction between the MeCP2 protein and the *Kcnj10* gene promoter in MeCP2 knockout mice. Supporting this notion, astrocyte cultures derived from MeCP2^–/y^ mice fail to upregulate Kir4.1 compared with cultures derived from WT mice. These results indicate that the loss of Kir4.1 is autonomous to the astrocyte and provide a mechanism for failure of Kir4.1 developmental upregulation in RTT mice. These data suggest that MeCP2 is a positive regulator of *Kcnj10* gene expression through development and potentially provide insight into how astrocytic dysfunction may contribute to RTT.

### The role of Kir4.1 in RTT and normal brain

Accumulating evidence indicates that Kir4.1 plays an integral role in the CNS. Decreased Kir4.1 currents are associated with various pathologies such as epilepsy, cerebral trauma, cerebral ischemia, cerebral inflammation, Alzheimer’s disease, amyotrophic lateral sclerosis, and Huntington’s disease (Nwaobi et al., 2016). Here, we demonstrate that astrocyte Kir4.1 currents are significantly reduced in symptomatic MeCP2-deficient mice. Of note, the extent to which astrocytes are clamped is uncertain because of the leaky membrane and the complex morphology. Reduction in Kir4.1 function may contribute to neuronal dysfunction. After firing of an action potential, K^+^ is extruded from neurons. Because of the small size of the extracellular space, small fluxes in K^+^ can lead to large concentration changes that are sufficient to modulate the efficacy of neuronal transmission (Ransom et al., 2000). Regulation of extracellular potassium ([K^+^]_o_) is the most well-recognized function of astrocytes (Kuffler and Potter, 1964; Orkand et al., 1966; Kuffler, 1967; Kofuji and Newman, 2004). Basal [K^+^]_o_ in rat hippocampal slices was increased when Kir channels were blocked with BaCl_2_ (D’Ambrosio et al., 2002). Loss of Kir4.1, with a similar increase in [K^+^]_o_, was reported in a mouse model of Huntington’s disease (Tong et al., 2014) and is associated with medium spiny neuron dysfunction. AAV-mediated rescue of Kir4.1 specifically to astrocytes rescued elevated K^+^, prolonged survival, and ameliorated motor deficits in these mice.

Our data demonstrate that loss of Kir4.1 in MeCP2-deficient mice is associated with an increase in [K^+^]_o_ and K^+^ undershoot (Fig. 2). Inhibition of Kir4.1 with 200 μM barium in rat hippocampus led to elevated [K^+^]_o_ and K^+^ undershoot (D’Ambrosio et al., 2002), as in our work. The undershoot under normal conditions is explained by a model in which Kir4.1 maintains [K^+^]_o_ by efflux of K^+^ from astrocytes to balance excessive K^+^ uptake by the ATPase (D’Ambrosio et al., 2002). Thus, loss of Kir4.1 leads to a larger K^+^ undershoot and perturbation of [K^+^]_o_.

Given the role that Kir channels play in the regulation of K^+^ concentration, loss of Kir4.1 in RTT may predict a change in neuronal excitability. There are conflicting reports regarding the excitability levels in the *Mecp2*-mutant brains. Some studies show hyperexcitability in various regions of mutant *Mecp2* mice from the level of the neuron (Zhang et al., 2010; Calfa et al., 2011b) up to the level of the neuronal network (Asaka et al., 2006; Moretti et al., 2006; Zhang et al., 2008; D’Cruz et al., 2010; Calfa et al., 2011b; Colic et al., 2011; McLeod et al., 2013). Other studies have not demonstrated the same hyperexcitability in RTT (Dani et al., 2005; Dani and Nelson, 2009; Wood et al., 2009). These discordant findings may be a result of examination at different stages of disease development. Studies reporting measurements from presymptomatic to early symptomatic animals (P14–P35) show hypoactivity, while studies demonstrating network hyperexcitability use tissue from older mice, which clearly exhibit symptoms associated with RTT (D’Cruz et al., 2010; Colic et al., 2011). Thus, excitability of cortical neurons may dramatically change during disease progression. Supporting this, measurements of cellular density in cortical layers II/III, IV, V, and VI in *Mecp2^–/y^* mice demonstrate that no abnormalities are observed at P14, but by P56 changes in all cortical layers are apparent (Kishi and Macklis, 2004). This fits the findings in this study, in which Kir4.1 expression is normal in early development, and thus [K^+^] regulation by astrocytes may not be disrupted. Failure to upregulate Kir4.1 expression in astrocytes may contribute to the hyperexcitability in aged, symptomatic animals.

Supporting the importance of Kir4.1 in normal brain function, patient populations who carry loss-of-function mutations in the *Kcnj10* gene have been identified. Intriguingly, CNS symptoms in these patients, including early-onset seizures, ataxia, profound lower motor extremity weakness, and severe cognitive deficits (Scholl et al., 2009; Sicca et al., 2011), are also commonplace in RTT patients. Of note, *Mecp2^–/y^* cortex demonstrated a significant reduction in Kir4.1 expression, not a complete loss, as would be the case in a *Kcnj10*-knockout animal. Although Kir4.1 currents are downregulated in *Mecp2^–/y^* mice, the resting membrane potential of the astrocytes appear unaltered. This may be due to other compensatory K^+^ channel regulation in astrocytes.

### Impaired developmental regulation of Kir4.1 in RTT mice

Kir4.1 protein and mRNA expression are strongly developmentally up-regulated in all brain regions, with highest expression observed in caudal brain structures (Nwaobi et al., 2014). Throughout the CNS, the increased expression of Kir4.1 parallels the increase in MeCP2 expression and coincides with the onset of symptoms in RTT. Upregulation of Kir4.1 is directly related to the methylation status of several CpG islands found in the promoter and intronic regions of the gene (Nwaobi et al., 2014). Furthermore, the *Kcnj10* promoter is methylated at a low level and highly expressed. Given that MeCP2 is a methyl-binding protein, which is often found bound to transcriptionally active genes with low-methylated promoters (Yasui et al., 2007; Chahrour et al., 2008), we speculated that MeCP2 was a positive transcriptional regulator of the Kir4.1 gene. Indeed, loss of MeCP2 leads to decreased Kir4.1 expression. Also, we found direct physical interaction between MeCP2 and a CpG-rich island in the promoter of *Kcnj10* using ChIP (Fig. 6). The data from the cultured isolated astrocytes (Fig. 5) suggests that loss of *Kcnj10* mRNA is autonomous to the astrocyte and not dependent on development in the diseased CNS. This is the first example of a glia-specific gene targeted by MeCP2 and suggests that MeCP2 positively regulates *Kcnj10* gene transcription.

We demonstrate that Kir4.1 expression does not undergo a typical robust developmental upregulation when MeCP2 is absent. Kir4.1 is not downregulated, but instead never reaches normal levels, suggesting that astrocytic maturation may be altered in the absence of MeCP2. *In vivo*, the loss of Kir4.1 that exists before the onset of severe symptomatology in *Mecp2*-deficient mice indicates that it may contribute to disease progression, and not simply an epiphenomenon of a “diseased” brain.

The vast majority of individuals with RTT are females, yet most animals studies focus on male *Mecp2* mutants. The reasons for this are that (1) male mice present with earlier symptoms and more severe symptoms, and (2) the loss of MeCP2 expression is less variable in male mice. Because the *Mecp2* gene is on the X chromosome, mutant female mice express one WT copy of *Mecp2*, leading to less severe and later symptoms. Additionally, random X chromosome inactivation may lead to skewed expression of the mutant or WT allele, which can cause variable disease progression. Nevertheless, given that RTT largely affects females, we investigated Kir4.1 mRNA expression in aged symptomatic female animals and show a loss of *Kcnj10* transcript similar to that observed in males.

### Kir4.1/*Kcnj10* dysregulation in Mecp2^–/y^ mice: from direct interaction to function

Transcriptomic and, more recently, proteomic studies have begun to shed light on gene and protein disruption in human patients and animal models of RTT (Colantuoni et al., 2001; Jordan et al., 2007; Chahrour et al., 2008; Urdinguio et al., 2008; Ben-Shachar et al., 2009; Gibson et al., 2010; Gabel et al., 2015; Gabel et al., 2015; Lin et al., 2016). Although no study has systematically evaluated astrocytes, a recent publication indicates that 46 of 391 differentially expressed genes are uniquely astrocytic, including Kir4.1 (Pacheco et al., 2017). The changes in astrocyte gene expression do not indicate a typical reactive gliosis as seen in most neurologic diseases, because in RTT tissue many markers associated with reactive gliosis are downregulated. Intriguingly, several disrupted genes are developmentally regulated, possibly indicating alterations in astrocyte maturation in the MeCP2-deficient cortex. Although numerous studies have demonstrated that MeCP2 has many neuronal targets, including Bdnf, Gabr3, Reln, Sst, and Creb1 (Singh et al., 2008), here we show for the first time that one such critical astrocytic gene, *Kcnj10*, is a direct molecular target of MeCP2.

Importantly, we also show the first electrophysiological studies performed in astrocytes indicating that normal membrane currents are affected in a mouse model of RTT. These data may shed light on the role of astrocytes in RTT and indicate that astrocytes and Kir4.1 may represent novel therapeutic targets for the treatment of RTT.

## Synthesis

Reviewing Editor: Tallie Z. Baram, University of California-Irvine Decisions are customarily a result of the Reviewing Editor and the peer reviewers coming together and discussing their recommendations until a consensus is reached. When revisions are invited, a fact-based synthesis statement explaining their decision and outlining what is needed to prepare a revision will be listed below. The following reviewer(s) agreed to reveal their identity: Tim Benke.

The manuscript in its revised form now presents convincing evidence to support down-regulation of Kir4.1 in astrocytes of MECP2-impaired animals, in an autonomous fashion. Considering the potential relevance to MECP2 impairment to Rett syndrome and the need to identify cellular pathways for potential therapeutic strategies, these findings should be of significant interest readership.

Thus, thee reviewers of the revised paper found it worthy of publication in eNeuro- and i concur heartily. One of the reviewers requested additional minor improvements, and i include these below. Upon completion of these, we will accept the paper. Thank you for working with us!

There are several remaining technical inconsistencies and inaccuracies that should be addressed:

1. Fig 4B: a p-value smaller than 0.05 is probably impossible to achieve with a 2-tailed unpaired t-test, based on the values reported (33% reduction with a standard error of 25%, see line 318 as well as the histogram). By entering these values into a statistical program (such as GraphPad Prism), one can see that even with a zero standard error of the control group (wildtype), the p-value would have still been ~0.2, way above the reported <0.05. The authors should clarify this discrepancy. If this is indeed statistically insignificant, the authors should modify the figure and text accordingly.

2. Line 294-295: "these higher molecular weight bands are absent in MECP2 deficient mice". Since higher bands are observable for these mice (Fig 3A), this statement should be altered to reflect reduced intensity rather than elimination of these bands.

3. Figure 5, legend (lines 689-690): "By 14 days in culture astrocytes express similar levels of astrocyte markers GFAP and Kir4.1 as observed in age-matched cortex". This is not supported by the data as presented (an increase is observed in Fig5B, which is expected considering that the cortex sample is of mixed cell populations). Unless the authors have data to support this statement , it should be removed.

4. Line 262: "TREK-2" - the authors have probably meant TREK-1 (KCNK2) (based on Table 2 and Results, line 242).

5. The methods section contains references to data that has been removed from the current version (e.g. lines 94-95, 112-113, describing antibodies and PCR probes used for various genes not included in this version).

6. Table 2: "Astros", "oligos" (right column) should be modified to their correct terms.

7. Line 253, 255: "not shown" - should be removed.

8. Figure 5 needs some work (panel letter B is misaligned, as well as "GFAP" title in panel A).

9. Fig.4 legend, line 683 "Mecp2 mice 2" -seems a typo.

## References

[B1] Asaka Y, Jugloff DGM, Zhang L, Eubanks JH, Fitzsimonds RM (2006) Hippocampal synaptic plasticity is impaired in the Mecp2-null mouse model of Rett syndrome. Neurobiol Dis 21:217–227. 10.1016/j.nbd.2005.07.005 16087343

[B2] Ballas N, Lioy DT, Grunseich C, Mandel G (2009) Non-cell autonomous influence of MeCP2-deficient glia on neuronal dendritic morphology. Nat Neurosci 12:311–317. 10.1038/nn.2275 19234456 PMC3134296

[B3] Batiuk MY, de Vin F, Duqué SI, Li C, Saito T, Saido T, Fiers M, Belgard TG, Holt MG (2017) An immunoaffinity-based method for isolating ultrapure adult astrocytes based on ATP1B2 targeting by the ACSA-2 antibody. J Biol Chem 292:8874–8891. 10.1074/jbc.M116.765313 28373281 PMC5448122

[B4] Ben-Shachar S, Chahrour M, Thaller C, Shaw CA, Zoghbi HY (2009) Mouse models of MeCP2 disorders share gene expression changes in the cerebellum and hypothalamus. Hum Mol Genet 18:2431–2442. 10.1093/hmg/ddp181 19369296 PMC2694691

[B5] Bockenhauer D, et al. (2009) Epilepsy, ataxia, sensorineural deafness, tubulopathy, and KCNJ10 mutations. N Engl J Med 360:1960–1970. 10.1056/NEJMoa0810276 19420365 PMC3398803

[B6] Bushong EA, Martone ME, Ellisman MH (2004) Maturation of astrocyte morphology and the establishment of astrocyte domains during postnatal hippocampal development. Int J Dev Neurosci 22:73–86. 10.1016/j.ijdevneu.2003.12.008 15036382

[B7] Calfa G, Percy AK, Pozzo-Miller L (2011a) Experimental models of Rett syndrome based on Mecp2 dysfunction. Exp Biol Med 236:3–19. 10.1258/ebm.2010.010261 PMC3059199 21239731

[B8] Calfa G, Hablitz JJ, Pozzo-Miller L (2011b) Network hyperexcitability in hippocampal slices from Mecp2 mutant mice revealed by voltage-sensitive dye imaging. J Neurophysiol 105:1768–1784. 21307327 10.1152/jn.00800.2010 PMC3075283

[B9] Chahrour M, Jung SY, Shaw C, Zhou X, Wong STC, Qin J, Zoghbi HY (2008) MeCP2, a key contributor to neurological disease, activates and represses transcription. Science 320:1224–1229. 10.1126/science.1153252 18511691 PMC2443785

[B10] Chen RZ, Akbarian S, Tudor M, Jaenisch R (2001) Deficiency of methyl-CpG binding protein-2 in CNS neurons results in a Rett-like phenotype in mice. Nat Genet 27:327–331. 10.1038/85906 11242118

[B11] Chever O, Djukic B, McCarthy KD, Amzica F (2010) Implication of Kir4.1 channel in excess potassium clearance: an in vivo study on anesthetized glial-conditional Kir4.1 knock-out mice. J Neurosci 30:15769–15777. 10.1523/JNEUROSCI.2078-10.2010 21106816 PMC6633770

[B12] Colantuoni C, Jeon OH, Hyder K, Chenchik A, Khimani AH, Narayanan V, Hoffman EP, Kaufmann WE, Naidu S, Pevsner J (2001) Gene expression profiling in postmortem rett syndrome brain: differential gene expression and patient classification. Neurobiology of Disease 8:847–865. 10.1006/nbdi.2001.0428 11592853

[B13] Colic S, Wither R, Eubanks JH, Zhang L, Bardakjian BL (2011) EEG analysis for estimation of duration and inter-event intervals of seizure-like events recorded in vivo from mice. Conf Proc IEEE Eng Med Biol Soc 2011:2570–2573. 10.1109/IEMBS.2011.6090710 22254866

[B14] Cuddapah VA, Pillai RB, Shekar KV, Lane JB, Motil KJ, Skinner SA, Tarquinio DC, Glaze DG, McGwin G, Kaufmann WE, Percy AK, Neul JL, Olsen ML (2014) Methyl-CpG-binding protein 2 (MECP2) mutation type is associated with disease severity in Rett syndrome. J Med Genet 51:152–158. 10.1136/jmedgenet-2013-102113 24399845 PMC4403764

[B15] D’Ambrosio R, Gordon DS, Winn HR (2002) Differential role of KIR channel and Na(+)/K(+)-pump in the regulation of extracellular K(+) in rat hippocampus. J Neurophysiol 87:87–102. 11784732 10.1152/jn.00240.2001

[B16] D’Cruz JA, Wu C, Zahid T, El-Hayek Y, Zhang L, Eubanks JH (2010) Alterations of cortical and hippocampal EEG activity in MeCP2-deficient mice. Neurobiol Dis 38:8–16. 20045053 10.1016/j.nbd.2009.12.018

[B17] Dani VS, Nelson SB (2009) Intact long-term potentiation but reduced connectivity between neocortical layer 5 pyramidal neurons in a mouse model of Rett syndrome. J Neurosci 29:11263–11270. 10.1523/JNEUROSCI.1019-09.2009 19741133 PMC2765053

[B18] Dani VS, Chang Q, Maffei A, Turrigiano GG, Jaenisch R, Nelson SB (2005) Reduced cortical activity due to a shift in the balance between excitation and inhibition in a mouse model of Rett syndrome. Proc Natl Acad Sci U S A 102:12560–12565. 10.1073/pnas.0506071102 16116096 PMC1194957

[B19] Derecki NC, Cronk JC, Lu Z, Xu E, Abbott SB, Guyenet PG, Kipnis J (2012) Wild-type microglia arrest pathology in a mouse model of Rett syndrome. Nature 484:105–109. 10.1038/nature10907 22425995 PMC3321067

[B20] Djukic B, Casper KB, Philpot BD, Chin LS, McCarthy KD (2007) Conditional knock-out of Kir4.1 leads to glial membrane depolarization, inhibition of potassium and glutamate uptake, and enhanced short-term synaptic potentiation. J Neurosci 27:11354–11365. 10.1523/JNEUROSCI.0723-07.2007 17942730 PMC6673037

[B21] Gabel HW, Kinde B, Stroud H, Gilbert CS, Harmin DA, Kastan NR, Hemberg M, Ebert DH, Greenberg ME (2015) Disruption of DNA-methylation-dependent long gene repression in Rett syndrome. Nature 522:89–93. 10.1038/nature14319 25762136 PMC4480648

[B22] Garg SK, Lioy DT, Knopp SJ, Bissonnette JM (2015) Conditional depletion of methyl-CpG-binding protein 2 in astrocytes depresses the hypercapnic ventilatory response in mice. J Appl Physiol 119:670–676. 10.1152/japplphysiol.00411.2015 26205541

[B23] Gibson JH, Slobedman B, KN H, Williamson SL, Minchenko D, El-Osta A, Stern JL, Christodoulou J (2010) Downstream targets of methyl CpG binding protein 2 and their abnormal expression in the frontal cortex of the human Rett syndrome brain. BMC Neurosci 11:53. 10.1186/1471-2202-11-53 20420693 PMC2881102

[B24] Glaze DG, Percy AK, Skinner S, Motil KJ, Neul JL, Barrish JO, Lane JB, Geerts SP, Annese F, Graham J, McNair L, Lee HS (2010) Epilepsy and the natural history of Rett syndrome. Neurology 74:909–912. 10.1212/WNL.0b013e3181d6b852 20231667 PMC2836870

[B25] Guy J, Hendrich B, Holmes M, Martin JE, Bird A (2001) A mouse Mecp2-null mutation causes neurological symptoms that mimic Rett syndrome. Nat Genet 27:322–326. 10.1038/85899 11242117

[B26] Holt LM, Olsen ML (2016) Novel applications of magnetic cell sorting to analyze cell-type specific gene and protein expression in the central nervous system. PLoS One 11:e0150290. 10.1371/journal.pone.0150290 26919701 PMC4769085

[B27] Jian L, Nagarajan L, de KN, Ravine D, Christodoulou J, Leonard H (2007) Seizures in Rett syndrome: an overview from a one-year calendar study. Eur J Paediatr Neurol 11:310–317. 10.1016/j.ejpn.2007.02.008 17433737 PMC3013620

[B28] Jordan C, Li HH, Kwan HC, Francke U (2007) Cerebellar gene expression profiles of mouse models for Rett syndrome reveal novel MeCP2 targets. BMC Med Genet 8:36. 10.1186/1471-2350-8-36 17584923 PMC1931432

[B29] Kantzer CG, Boutin C, Herzig ID, Wittwer C, Reiß S, Tiveron MC, Drewes J, Rockel TD, Ohlig S, Ninkovic J, Cremer H, Pennartz S, Jungblut M, Bosio A (2017) Anti-ACSA-2 defines a novel monoclonal antibody for prospective isolation of living neonatal and adult astrocytes. Glia 65:990–1004. 10.1002/glia.23140 28317180

[B30] Kifayathullah LA, Arunachalam JP, Bodda C, Agbemenyah HY, Laccone FA, Mannan AU (2010) MeCP^2270^ mutant protein is expressed in astrocytes as well as in neurons and localizes in the nucleus. Cytogenet Genome Res 129:290–297. 10.1159/000315906 20625242

[B31] Kishi N, Macklis JD (2004) MECP2 is progressively expressed in post-migratory neurons and is involved in neuronal maturation rather than cell fate decisions. Mol Cell Neurosci 27:306–321. 10.1016/j.mcn.2004.07.006 15519245

[B32] Kofuji P, Newman EA (2004) Potassium buffering in the central nervous system. Neuroscience 129:1043–1054. 10.1016/j.neuroscience.2004.06.008 PMC2322935 15561419

[B33] Kofuji P, Ceelen P, Zahs KR, Surbeck LW, Lester HA, Newman EA (2000) Genetic inactivation of an inwardly rectifying potassium channel (Kir4.1 subunit) in mice: phenotypic impact in retina. J Neurosci 20:5733–5740. 10908613 10.1523/JNEUROSCI.20-15-05733.2000 PMC2410027

[B34] Kuffler SW (1967) The Ferrier Lecture: Neuroglial cells: physiological properties and a potassium mediated effect of neuronal activity on the glial membrane potential. Proc R Soc Lond Ser B Biol Sci 168:1–21. 10.1098/rspb.1967.0047 4382871

[B35] Kuffler SW, Potter DD (1964) GLIA in the leech central nervous system: physiological properties and neuron-GLIA relationship. J Neurophysiol 27:290–320. 10.1152/jn.1964.27.2.290 14129773

[B36] Li W, Calfa G, Larimore J, Pozzo-Miller L (2012) Activity-dependent BDNF release and TRPC signaling is impaired in hippocampal neurons of Mecp2 mutant mice. Proc Natl Acad Sci U S A 109:17087–17092. 10.1073/pnas.1205271109 23027959 PMC3479462

[B37] Lin P, Nicholls L, Assareh H, Fang Z, Amos TG, Edwards RJ, Assareh AA, Voineagu I (2016) Transcriptome analysis of human brain tissue identifies reduced expression of complement complex C1Q genes in Rett syndrome. BMC Genomics 17:427. 10.1186/s12864-016-2746-7 27267200 PMC4895974

[B38] Lioy DT, Garg SK, Monaghan CE, Raber J, Foust KD, Kaspar BK, Hirrlinger PG, Kirchhoff F, Bissonnette JM, Ballas N, Mandel G (2011) A role for glia in the progression of Rett’s syndrome. Nature 475:497–500. 10.1038/nature10214 21716289 PMC3268776

[B39] Liu F, Ni J-J, Sun F-Y (2017) Expression of phospho-MeCP2s in the developing rat brain and function of postnatal MeCP2 in cerebellar neural cell development. Neurosci Bull 33:1–16. 10.1007/s12264-016-0086-x 27995568 PMC5567549

[B40] Liu F, Ni J-J, Huang J-J, Kou Z-W, Sun F-Y (2015) VEGF overexpression enhances the accumulation of phospho-S292 MeCP2 in reactive astrocytes in the adult rat striatum following cerebral ischemia. Brain Res 1599:32–43. 10.1016/j.brainres.2014.12.014 25511996

[B41] Ma B, Xu G, Wang W, Enyeart JJ, Zhou M (2014) Dual patch voltage clamp study of low membrane resistance astrocytes in situ. Mol Brain 7:18. 10.1186/1756-6606-7-18 24636341 PMC3995526

[B42] Maezawa I, Jin LW (2010) Rett syndrome microglia damage dendrites and synapses by the elevated release of glutamate. J Neurosci 30:5346–5356. 10.1523/JNEUROSCI.5966-09.2010 20392956 PMC5533099

[B43] Maezawa I, Swanberg S, Harvey D, LaSalle JM, Jin LW (2009) Rett syndrome astrocytes are abnormal and spread MeCP2 deficiency through gap junctions. J Neurosci 29:5051–5061. 10.1523/JNEUROSCI.0324-09.2009 19386901 PMC3436907

[B44] McLeod F, Ganley R, Williams L, Selfridge J, Bird A, Cobb SR (2013) Reduced seizure threshold and altered network oscillatory properties in a mouse model of Rett syndrome. Neuroscience 231:195–205. 10.1016/j.neuroscience.2012.11.058 23238573

[B45] Moretti P, Levenson JM, Battaglia F, Atkinson R, Teague R, Antalffy B, Armstrong D, Arancio O, Sweatt JD, Zoghbi HY (2006) Learning and memory and synaptic plasticity are impaired in a mouse model of Rett syndrome. J Neurosci 26:319–327. 10.1523/JNEUROSCI.2623-05.2006 16399702 PMC6674314

[B46] Nectoux J, Florian C, Delepine C, Bahi-Buisson N, Khelfaoui M, Reibel S, Chelly J, Bienvenu T (2012) Altered microtubule dynamics in Mecp2-deficient astrocytes. J Neurosci Res 90:990–998. 10.1002/jnr.23001 22252744

[B47] Neul JL, Kaufmann WE, Glaze DG, Christodoulou J, Clarke AJ, Bahi-Buisson N, Leonard H, Bailey ME, Schanen NC, Zappella M, Renieri A, Huppke P, Percy AK (2010) Rett syndrome: revised diagnostic criteria and nomenclature. Ann Neurol 68:944–950. 10.1002/ana.22124 21154482 PMC3058521

[B48] Neusch C, Papadopoulos N, Müller M, Maletzki I, Winter SM, Hirrlinger J, Handschuh M, Bähr M, Richter DW, Kirchhoff F, Hülsmann S (2006) Lack of the Kir4.1 channel subunit abolishes K+ buffering properties of astrocytes in the ventral respiratory group: impact on extracellular K+ regulation. J Neurophysiol 95:1843–1852. 10.1152/jn.00996.2005 16306174

[B49] Nwaobi SE, Lin E, Peramsetty SR, Olsen ML (2014) DNA methylation functions as a critical regulator of Kir4.1 expression during CNS development. Glia 62:411–427. 10.1002/glia.22613 24415225 PMC3991476

[B50] Nwaobi SE, Cuddapah VA, Patterson KC, Randolph AC, Olsen ML (2016) The role of glial-specific Kir4.1 in normal and pathological states of the CNS. Acta Neuropathol 132:1–21. 10.1007/s00401-016-1553-1 26961251 PMC6774634

[B51] Okabe Y, Takahashi T, Mitsumasu C, Kosai K, Tanaka E, Matsuishi T (2012) Alterations of gene expression and glutamate clearance in astrocytes derived from an MeCP2-null mouse model of Rett syndrome. PLoS One 7:e35354. 10.1371/journal.pone.0035354 22532851 PMC3332111

[B52] Olsen ML, Higashimori H, Campbell SL, Hablitz JJ, Sontheimer H (2006) Functional expression of Kir4.1 channels in spinal cord astrocytes. Glia 53:516–528. 10.1002/glia.20312 16369934 PMC2553202

[B53] Orkand RK, Nicholls JG, Kuffler SW (1966) Effect of nerve impulses on the membrane potential of glial cells in the central nervous system of amphibia. J Neurophysiol 29:788–806. 10.1152/jn.1966.29.4.788 5966435

[B54] Percy AK (2002) Rett syndrome. Current status and new vistas. Neurol Clin 20:1125–1141. 12616684 10.1016/s0733-8619(02)00022-1

[B55] Ransom CB, Sontheimer H (1995) Biophysical and pharmacological characterization of inwardly rectifying K+ currents in rat spinal cord astrocytes. J Neurophysiol 73:333–346. 10.1152/jn.1995.73.1.333 7714576

[B56] Ransom CB, Ransom BR, Sontheimer H (2000) Activity-dependent extracellular K+ accumulation in rat optic nerve: the role of glial and axonal Na+ pumps. J Physiol 522:427–442. 10.1111/j.1469-7793.2000.00427.x 10713967 PMC2269766

[B57] Sajan SA, Jhangiani SN, Muzny DM, Gibbs RA, Lupski JR, Glaze DG, Kaufmann WE, Skinner SA, Annese F, Friez MJ, Lane J, Percy AK, Neul JL (2017) Enrichment of mutations in chromatin regulators in people with Rett syndrome lacking mutations in MECP2. Genet Med 19:13–19. 10.1038/gim.2016.42 27171548 PMC5107176

[B58] Scholl UI, Choi M, Liu T, Ramaekers VT, Häusler MG, Grimmer J, Tobe SW, Farhi A, Nelson-Williams C, Lifton RP (2009) Seizures, sensorineural deafness, ataxia, mental retardation, and electrolyte imbalance (SeSAME syndrome) caused by mutations in KCNJ10. Proc Natl Acad Sci U S A 106:5842–5847. 10.1073/pnas.0901749106 19289823 PMC2656559

[B59] Seifert G, Hüttmann K, Binder DK, Hartmann C, Wyczynski A, Neusch C, Steinhauser C (2009) Analysis of astroglial K+ channel expression in the developing hippocampus reveals a predominant role of the Kir4.1 subunit. J Neurosci 29:7474–7488. 10.1523/JNEUROSCI.3790-08.2009 19515915 PMC6665420

[B60] Sicca F, Imbrici P, D’Adamo MC, Moro F, Bonatti F, Brovedani P, Grottesi A, Guerrini R, Masi G, Santorelli FM, Pessia M (2011) Autism with seizures and intellectual disability: possible causative role of gain-of-function of the inwardly-rectifying K+ channel Kir4.1. Neurobiol Dis 43:239–247. 10.1016/j.nbd.2011.03.016 21458570

[B61] Singh J, Saxena A, Christodoulou J, Ravine D (2008) MECP2 genomic structure and function: insights from ENCODE. Nucleic Acids Res 36:6035–6047. 10.1093/nar/gkn591 18820302 PMC2577328

[B62] Smrt RD, Pfeiffer RL, Zhao X (2011) Age-dependent expression of MeCP2 in a heterozygous mosaic mouse model. Hum Mol Genet 20:1834–1843. 10.1093/hmg/ddr066 21330301 PMC3071674

[B63] Tong X, Ao Y, Faas GC, Nwaobi SE, Xu J, Haustein MD, Anderson MA, Mody I, Olsen ML, Sofroniew MV, Khakh BS (2014) Astrocyte Kir4.1 ion channel deficits contribute to neuronal dysfunction in Huntington’s disease model mice. Nat Neurosci 17:694–703. 10.1038/nn.3691 24686787 PMC4064471

[B64] Urdinguio RG, Lopez-Serra L, Lopez-Nieva P, Alaminos M, Diaz-Uriarte R, Fernandez AF, Esteller M (2008) Mecp2-null mice provide new neuronal targets for Rett syndrome. PLoS One 3:e3669. 10.1371/journal.pone.0003669 18989361 PMC2576441

[B65] Williams EC, Zhong X, Mohamed A, Li R, Liu Y, Dong Q, Ananiev GE, Mok JCC, Lin BR, Lu J, Chiao C, Cherney R, Li H, Zhang S-C, Chang Q (2014) Mutant astrocytes differentiated from Rett syndrome patients-specific iPSCs have adverse effects on wild-type neurons. Hum Mol Genet 23:2968–2980. 10.1093/hmg/ddu008 24419315 PMC4014193

[B66] Wood L, Gray NW, Zhou Z, Greenberg ME, Shepherd GMG (2009) Synaptic circuit abnormalities of motor-frontal layer 2/3 pyramidal neurons in an RNA interference model of methyl-CpG-binding protein 2 deficiency. J Neurosci 29:12440–12448. 10.1523/JNEUROSCI.3321-09.2009 19812320 PMC2782478

[B67] Yasui DH, Peddada S, Bieda MC, Vallero RO, Hogart A, Nagarajan RP, Thatcher KN, Farnham PJ, Lasalle JM (2007) Integrated epigenomic analyses of neuronal MeCP2 reveal a role for long-range interaction with active genes. Proc Natl Acad Sci U S A 104:19416–19421. 10.1073/pnas.0707442104 18042715 PMC2148304

[B68] Zachariah RM, Olson CO, Ezeonwuka C, Rastegar M (2012) Novel MeCP2 isoform-specific antibody reveals the endogenous MeCP2E1 expression in murine brain, primary neurons and astrocytes. PLoS One 7:e49763. 10.1371/journal.pone.0049763 23185431 PMC3501454

[B69] Zhang L, He J, Jugloff DG, Eubanks JH (2008) The MeCP2-null mouse hippocampus displays altered basal inhibitory rhythms and is prone to hyperexcitability. Hippocampus 18:294–309. 10.1002/hipo.20389 18058824

[B70] Zhang X, Cui N, Wu Z, Su J, Tadepalli JS, Sekizar S, Jiang C (2010) Intrinsic membrane properties of locus coeruleus neurons in Mecp2-null mice. Am J Physiol Cell Physiol 298:C635–C646. 10.1152/ajpcell.00442.2009 20042730 PMC2838567

[B71] Zheng H, Stornetta RL, Agassandian K, Rinaman L (2014) Glutamatergic phenotype of glucagon-like peptide 1 neurons in the caudal nucleus of the solitary tract in rats. Brain Struct Funct 220:3011–3022. 25012114 10.1007/s00429-014-0841-6 PMC5330389

[B72] Zhou M, Schools GP, Kimelberg HK (2006) Development of GLAST(+) astrocytes and NG2(+) glia in rat hippocampus CA1: mature astrocytes are electrophysiologically passive. J Neurophysiol 95:134–143. 10.1152/jn.00570.2005 16093329

